# Translating, Adapting and Validating the Revised MISSCARE Survey for Use in Norwegian Hospitals—A Pilot Study

**DOI:** 10.1177/23779608251332742

**Published:** 2025-04-04

**Authors:** Kjersti Grønning, Melliane Muteba Olsen, Beate André

**Affiliations:** 1 Department of Research, 60500 Nord-Trøndelag Hospital Trust, Levanger, Norway; 2 Department of Public Health and Nursing, 8018 Norwegian University of Science and Technology, Trondheim, Norway

**Keywords:** pilot study, nursing, questionnaire, validation

## Abstract

**Background:**

The original *MISSCARE Survey* was developed in the US in the early 2000s to assess the amount of missed nursing care. Because additional causes of missed care were detected in later years, the *MISSCARE Survey* was further developed in 2019 by adding one item in Part A and five items in Part B to the questionnaire. Neither the original nor the *revised MISSCARE Survey* is translated into Norwegian, so a questionnaire is needed to assess missed nursing care in Norway. This study aims to translate and adapt the revised *MISSCARE Survey* for use in Norwegian hospitals.

**Methods:**

A forward translation, followed by an expert panel's back-translation, cognitive interviews, and final version testing were conducted. Exploratory factor analyses were conducted to investigate the underlying factor structure. Internal consistency was assessed using Cronbach's alpha, and the intraclass correlation coefficient (ICC) was employed for a test–retest evaluation. IBM SPSS Statistics (version 29) was used for all analyses.

**Results:**

A total of 120 nurses and nursing assistants took part in the study assessing the psychometric properties of the Norwegian adaptation of the revised *MISSCARE Survey*. The exploratory factor analysis for Part B revealed four factors, the Kaiser–Meyer–Olkin measure of sampling adequacy was 0.895, and Cronbach's alpha values ranged from 0.841 to 0.751, reflecting good internal consistency. The overall test–retest ICC was 0.894 for Part A and 0.827 for Part B, indicating strong reliability.

**Conclusions:**

The revised *MISSCARE Survey* adapted for use in Norwegian hospitals is a reliable and promising instrument for assessing missed nursing care in medical and surgical units within a local Norwegian hospital. However, further studies should be conducted to confirm the factor structure in larger and more diverse populations.

## Introduction

Missed nursing care, also known as care left undone, refers to care that is delayed, only partially completed, or not completed at all (Kalisch et al., 2009). Incomplete nursing care involves a problem (insufficient resources or time), a process (clinical decision-making to prioritize and ration care), and an outcome (unfinished care) (Jones et al., 2015; Kalánková et al., 2019). Missed, rationed, or unfinished nursing care is a significant concern due to its lack of comprehensive understanding, highlighting the need for further exploration (Kalánková et al., 2019).

### Review of the Literature

Several studies show many negative consequences when the care is left undone or missed (Ball et al., 2018; Duffy et al., 2018; Gathara et al., 2020; Kalisch & Lee, 2012; Kalisch et al., 2012a, 2012b) and is therefore a threat to patient safety (Ball et al., 2018; Chaboyer et al., 2021; Kalánková et al., 2019; Kalisch & Lee, 2012; Liu et al., 2018; Peterson et al., 2022; Recio-Saucedo et al., 2018). Reported negative consequences associated with missed care are patient falls (Kalisch & Lee, 2012; Kalisch et al., 2012b), prolonged hospital stays and delayed discharges, heightened pain and discomfort, physical disabilities (Duffy et al., 2018), reduced quality of life, and a higher incidence of pneumonia, urinary tract infections, sepsis, medical management errors, pressure ulcers, and hospital-acquired infections (Ball et al., 2018; Kalisch et al., 2012a, 2012b). The World Health Organization (WHO) reports that unsafe care results in the loss of 64 million disability-adjusted life years annually worldwide (WHO, 2021).

The *MISSCARE survey* was created to identify nursing tasks that were either partially or entirely omitted, or delayed, and to understand the circumstances leading to incomplete care in the United States during the early 2000s (Kalisch, 2006; Kalisch & Williams, 2009). Numerous studies have identified the primary causes of missed nursing care as staffing shortages (Ball et al., 2014; Chaboyer et al., 2021; Danielis et al., 2021), inadequate material resources, poor communication (Chaboyer et al., 2021; Dutra & Guirardello, 2021; Saqer & AbuAlRub, 2018), excessive workload (Chaboyer et al., 2021; Du et al., 2020), lack of readiness for unforeseen situations, challenges in a suboptimal work environment, and ineffective organizational planning (Andersson et al., 2022). Studies also indicate that missed or incomplete nursing care is primarily attributed to time constraints (Kalánková et al., 2019), and that planning and communication are more frequently reported as missed care compared to clinical procedures and patient monitoring/observation (Griffiths et al., 2018).

Since Beatrice Kalisch and her team developed the *MISSCARE survey* in the early 2000s (Kalisch & Williams, 2009), the questionnaire has been translated from US English into many different languages (Bragadóttir et al., 2015; Danielis et al., 2021; Kalisch et al., 2012a; Lisby et al., 2023; Nymark et al., 2020; Siqueira et al., 2013). The *MISSCARE Survey* was further revised in 2019 because additional causes of missed care were detected (Dabney et al., 2019). Neither the original nor the revised *MISSCARE Survey* is translated into Norwegian. Several studies emphasize the necessity of gaining deeper insights into the reasons for missed nursing care (Fennelly et al., 2021; Kim et al., 2017; Papathanasiou et al., 2024; Patterson et al., 2017). Numerous studies also show that the *MISSCARE survey* is a reliable and valid tool for evaluating clinical nursing practice and understanding the reasons behind missed care (Bragadóttir et al., 2015; Danielis et al., 2021; Kalisch et al., 2012a; Lisby et al., 2023; Nymark et al., 2020; Siqueira et al., 2013). In comparison to other available tools for measuring the frequency or prevalence of unfinished care in hospitals, the *MISSCARE survey* is the only measure designed to address the causes of missed care, making it possible to identify its antecedents, designing actions and evaluate their effectiveness (Palese et al., 2021). This study therefore aims to translate and adapt the revised *MISSCARE Survey* for use in Norwegian hospitals.

## Methods

The process of translation, adaptation, and validation of the Norwegian version of the revised *MISSCARE Survey* followed international guidelines for translating, adaptation and validation of instruments (Cruchinho et al., 2024; Sousa & Rojjanasrirat, 2011), including initial translation, back-translation, expert committee review, cognitive interviewing, validation, and evaluation of psychometric properties.

### The Revised MISSCARE Survey

Part A in the revised *MISSCARE Survey* contains 25 items (nursing care tasks) (Dabney et al., 2019) compared to 24 in the original questionnaire (Kalisch & Williams, 2009). The respondents are requested to indicate how often each task is missed in their unit using a 5-point Likert scale. The response options range from “always missed” to “never missed”. Part B of the revised version (Dabney et al., 2019) includes 22 items, an increase from the 17 items in the original version (Kalisch & Williams, 2009). These items address reasons for missed nursing care. The response options are presented on a 4-point Likert scale, ranging from “a significant reason” to “not a reason” for missed nursing care (Dabney et al., 2019).

### The Translation Process

A professional translation company conducted the forward translation of the questionnaire. The researchers (KG and BA), both registered nurses (RNs) with PhDs, thoroughly reviewed the translations and identified expressions and other discrepancies that needed to be resolved. The preliminary translated version was reviewed by an expert panel consisting of four RNs from the hospital: one holding a PhD, one with a master's degree, and two with bachelor's degrees. After receiving feedback from the expert panel, a consensus was made on a preliminary version. Then, two independent translators with PhDs, one RN and one health science researcher, performed the back-translation from Norwegian to English. The translators were blind to the original version.

To validate the back-translation against the original version, three clinically experienced RNs, one professor, and two PhD candidates were asked to evaluate each item individually. They were instructed to indicate whether they perceived the translation as having “exactly the same meaning” (both content and wording were identical), “almost the same meaning” (content was the same but wording differed), or “different meaning” (both content and wording differed). To assess the face validity of the questionnaire, the first author held two meetings with RNs working in the clinic, during which the items were presented. The nurses were encouraged to comment if the questions were unclear, or if there were phrasings they did not understand. We also invited eight undergraduate nursing students, working as nursing assistants at the hospital, to comment if the questions were uncertain or if there were expressions, they did not comprehend. In the final step, after reviewing the back-translation ratings along with the face validity testing results, the research team agreed on a final Norwegian version of the revised *MISSCARE Survey*. This version included some modifications, clarifications, or refinements to fit the Norwegian context and culture better, described in the supplementary file “Modifications of the Norwegian version of the revised MISSCARE Survey.” The response alternative “not relevant” for Part A was also added as an option because some of the listed nursing care activities are irrelevant for some hospitalized patients in some care contexts in Norwegian hospitals.

### The Evaluation of the Psychometric Properties of the Norwegian Version of the Revised *MISSCARE Survey*

#### Participants

The inclusion criteria for the study evaluating the psychometric properties of the Norwegian version of the revised *MISSCARE Survey* were RNs and nursing assistants working in a medical or surgical department offering 24-7 care at a local hospital in Mid-Norway. The hospital has approximately 90 medical and surgical beds. The exclusion criteria were RNs and nursing assistants working in outpatient departments and the intensive care unit. From October to November 2022, a sample of 193 eligible RNs and nursing assistants were invited to participate in the study. The participants answered the questionnaire on two occasions as a test and retest, with 2 to 3 weeks between the first and the second invitation. The questionnaire was sent to each participant's email address at the hospital. One reminder was sent to the participants.

### Statistical Analysis

The statistical analyses were conducted using SPSS, version 29. The study population's characteristics are presented in terms of numbers and proportions. Acceptability is measured by the number and proportion of missing responses for each of the items in Part A (nursing care tasks) and Part B (reasons for missed nursing care tasks). Construct validity for Part B was assessed using exploratory factor analysis (EFA) with each potential factor representing an underlying construct of missed care. The purpose of EFA is to uncover the latent (unobserved) structures or factors that explain the patterns of correlations among observed variables. Factor loadings of more than 0.3 are acceptable, communalities should exceed 0.2, and the Kaiser–Meyer–Olkin (KMO) measure should be greater than 0.6. Reliability was evaluated through standard measures of internal consistency and test–retest reliability, such as Cronbach's alpha coefficient and the intraclass correlation coefficient (ICC) (Polit & Beck, 2012). Cronbach's Alpha calculates the average correlation among items in a scale, where a higher alpha value (above 0.70) indicates good internal consistency. The ICC value ranges from 0 to 1, where higher values indicate greater reliability. Values less than 0.50 are considered poor reliability, while values from 0.50 to 0.75 and from 0.75 to 0.90 are considered as moderate and good reliability, respectively. Values greater than 0.90 are excellent reliability.

### Ethical Considerations

The participants received written information about the purpose of the study, highlighting that their involvement was voluntary and that completing the web-based questionnaire implied their consent. No directly identifiable personal or sensitive data was collected. The Data Access Committee (DAC) at Nord-Trøndelag Hospital Trust (DAC ref. 2022_2333) approved all procedures related to the handling and storage of personal data, in accordance with Norwegian legislation (Act on the Processing of Personal Data, 2022; The Norwegian Health Research Act, 2021).

## Results

**Figure 1.**
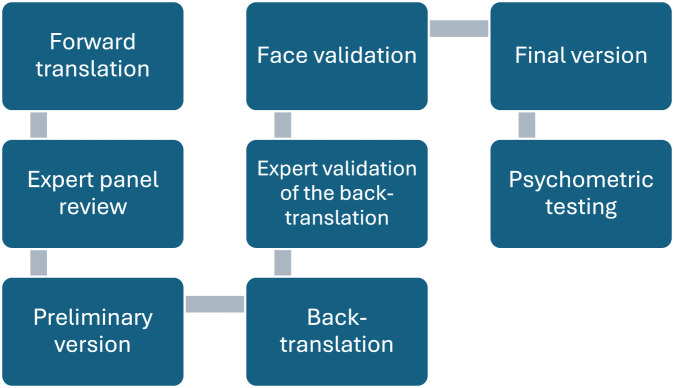
The translation and validation process.

Figure 1 The translation and validation process illustrates the process of translating, adapting, and validating the revised *MISSCARE Survey* in Norwegian.

### Demographics

We received responses from 120 out of the 193 eligible participants, resulting in a response rate of 62.2%. Table 1 provides an overview of the study sample's characteristics. There were 103 RNs and 13 nursing assistants. Four participants indicated they had a different role but did not specify the nature of that role. Approximately 80% of the participants were under 55 years old, with the majority being female. Around 80% held a bachelor's degree, and 90% worked in shifts. Over half of the sample had an overall work experience of 10 years or more, and approximately 40% had over a decade of experience in their current unit.

**Table 1.** Sample Characteristics.

Characteristics	N = 120
Role (%)	
Registered nurses	103 (86)
Nurse assistants	13 (11)
Other	4 (3)
Wards (%)	
Surgical	46 (38)
Medical	71 (59)
Other	3 (3)
Highest level of education (%)	
Primary school	-
High school	12 (10)
Bachelor's degree	97 (80)
Master's degree or higher	10 (8)
Sex (%)	
Female	103 (86)
Male	16 (13)
Age groups (%)	
<25 years	9 (8)
25–34	30 (25)
35–44	24 (20)
45–54	33 (28)
55–64	21 (18)
65 +	2 (2)
Working hours per week (%)	
<30	24 (20)
≥30	95 (79)
Work-shifts (%)	
Only daytime	12 (10)
Nightshifts	7 (6)
Day /afternoon shifts	29 (24)
Day/afternoon/night shifts	71 (59)

### Acceptability

The proportion of participants who fully completed the survey, without leaving any items blank, was 95.8% for Part A and 90.8% for Part B. No participants left either Part A or B completely blank.

### Construct Validity

Cases with missing data were excluded from the EFA for Part B. The EFA revealed a KMO measure of sampling adequacy of 0.895, suggesting that the correlation patterns are fairly tight. Bartlett's test of sphericity was significant, with a p-value less than 0.001. Factors were extracted based on eigenvalues greater than 1.0, and the analysis indicated a four-factor solution, as shown in Table 2 below. The factor loadings ranged from 0.483 to 0.737 which is considered acceptable. However, the two items with loadings close to 0.4 (items 4 and 16) may indicate that these items are less strongly related to factors 1 and 2, respectively. Furthermore, each item showed sufficient loading on at least one factor; however, 12 items (items 1, 4, 9, 10, 12, 13, 14, 15, 16, 17, 19, and 20) exhibited loadings on two factors. The items’ wording in the revised *MISSCARE Survey* as presented in Table 2 has been condensed to keywords. The precise phrasing can be found in the original and revised *MISSCARE Survey* papers (Dabney et al., 2019; Kalisch & Williams, 2009).

**Table 2.** Factor Structure and Internal Consistency.

Factor	Cronbach's α	Items		Mean	SD	Factor loadings
**1**	.841	6	Medications unavailable	3.04	.728	0.737
		8	Care not provided by other departments	3.06	.701	0.678
		10	Supplies/equipment malfunction	3.18	.687	0.647
		7	Inadequate hand-off	3.03	.607	0.630
		19	Inadequate supervision of nursing assistants	3.33	.649	0.547
		9	Supplies/equipment unavailable	2.95	.708	0.541
		4	Inadequate assistive/clerical personnel	2.35	1.071	0.483
**2**	.834	22	Inadequate leadership support	3.13	.844	0.781
		18	Emotional/physical exhaustion	2.82	.903	0.594
		21	Lack of cues/reminders	3.15	.647	0.591
		16	Caregiver unavailable	2.89	.787	0.459
		17	Heavy admission/discharge activity	2.13	.960	0.552
		5	Unbalanced patient assignments	2.51	.870	0.540
		20	Interruptions/Multitasking	2.31	.921	0.503
**3**	.817	14	Tension with medical staff	2.73	.816	0.676
		13	Tension within nursing team	3.21	.621	0.660
		12	Tension with ancillary/support departments	3.27	.600	0.621
		11	Lack of team backup	3.23	.735	0.581
		15	Nursing assistant communication failure	3.17	.696	0.536
**4**	.751	2	Urgent patient situations	2.07	.908	0.812
		3	Unexpected patient volume/acuity	1.64	.826	0.783
		1	Inadequate staff	1.71	.834	0.622

Test–retest reliability was evaluated with a sample of 33 participants. The overall test–retest ICC was 0.894 for Part A and 0.827 for Part B, indicating strong reliability. The Cronbach's alpha values for the four factors ranged from 0.841 to 0.751, reflecting good internal consistency, as shown in Table 2.

## Discussion

Several studies have emphasized that we need a deeper insight into reasons for missed nursing care (Fennelly et al., 2021; Kim et al., 2017; Papathanasiou et al., 2024; Patterson et al., 2017). To the best of our knowledge, there is no reliable and valid Norwegian tool available for assessing clinical nursing practice and understanding the reasons for missed care. Since The *MISSCARE Survey* has been translated into many languages and found to be a valid and reliable tool for assessing missed nursing care, this study aimed to translate and adapt the revised *MISSCARE Survey* (Dabney et al., 2019) for use in Norwegian hospitals. When following international guidelines for translating, adapting, and validating instruments (Cruchinho et al., 2024; Sousa & Rojjanasrirat, 2011), we discovered that translating and adapting the US-developed *MISSCARE Survey* for use in Norwegian hospitals was challenging. During the translation process, we needed to make some linguistic changes, modifications, clarifications, or refinements to make the questionnaire appropriate for the cultural context of Norway. Other Nordic researchers (Bragadóttir et al., 2015; Lisby et al., 2023; Nymark et al., 2020) have also reported some challenges when translating the *MISSCARE Survey* into Icelandic, Danish, and Swedish. For instance, the term “nursing assistant” used in item 15 Part B “Nursing assistant did not communicate that care was not provided” did not apply to the Danish way of organizing nursing in hospital units (Lisby et al., 2023). Finding equivalent words, both in content and semantic terms in some items was also described as challenging when translating the questionnaire into Swedish (Nymark et al., 2020), while Bragadottir and co-workers found that missed nursing care was a relatively new concept in nursing, and Icelandic nurses were unfamiliar with it (Bragadóttir et al., 2015). This brought several challenges when translating the *MISSCARE Survey* into Icelandic.

Nevertheless, the main finding of this study was that the Norwegian adaptation of the revised *MISSCARE Survey* is a reliable and promising instrument for assessing missed nursing care in medical and surgical units in Norwegian hospitals. Unlike the findings of Dabney and colleagues (Dabney et al., 2019), our analysis revealed that Part B of the Norwegian version of the revised *MISSCARE Survey* is composed of four factors, while the original *MISSCARE survey* (Kalisch & Williams, 2009) and the revised version (Dabney et al., 2019) show that Part B is composed of three factors. The first factor pertains to the quality and level of communication within the healthcare team, the second factor relates to labor resources including staffing and multitasking, and the third factor involves material resources such as supplies and medications (Dabney et al., 2019; Kalisch & Williams, 2009).

The EFA analyses in this study as presented in Table 2 show that items 6, 8,10, 7,19, 9, and 4 are included in factor one, which we interpret as the level and quality of external resources and supplies. The items assess whether there is a lack of essential supplies and equipment, whether the hand-offs and supervision of nursing assistants and assistive/clerical staff are insufficient, and whether care is provided by other departments. The seven items 22, 18, 21, 16, 17, 5, and 20 encompass factor two and reflect the quality and extent of necessary internal management support as these items target leadership support, workload, interruptions and the availability of caregivers. Items 14, 13, 12, 11, and 15 are included in factor three, representing the quality of communication and collaboration. These items address tensions and communicative challenges with the medical staff, nursing team, and ancillary/support departments. Lastly, factor four includes items 2, 3, and 1, which we interpret as indicating critical work overload or care burden.

The differences in the factor structure between the Norwegian and US versions of the revised *MISSCARE Survey* (Dabney et al., 2019) could be due to several factors. One possible explanation is the cultural variations in the organization of hospital care between Norway and the US (Øyri et al., 2023). Norway's universal healthcare system is funded through taxes, emphasizing equal access to healthcare services for all citizens and prioritizing preventive care. This may influence the types of missed care reported and the factors identified in the survey. Furthermore, because nursing care is shaped by cultural contexts, the validity and reliability of the questionnaire might vary depending on the culture (Cao et al., 2023). Given that this study identified an alternative factor structure in the revised *MISSCARE Survey* (Dabney et al., 2019), more studies are needed to gain deeper knowledge about the factor structure. The anticipated three-factor model in the Swedish study was not validated by confirmatory factor analysis (CFA) either (Nymark et al., 2020).

### Strengths and Limitations

One strength of this study is that it is the first study aimed to translate and adapt a measure for assessing missed nursing care in Norway. Even though this study did not achieve excellent scores for cross-cultural validity, and our study sample was relatively small, the sample is above the threshold for conducting EFA analyses (Cruchinho et al., 2024). Part B in the revised *MISSCARE Survey* consists of 22 items, and the minimum sample size is five to 10 individuals per item. Thus, a higher ratio increases the likelihood of achieving a strong factor structure model. However, there are some limitations. One limitation is that the study was conducted in a single local hospital within the health region, leading to a limited sample size (N = 120). The small sample size did not allow us to conduct confirmatory factor analyses (CFA) to potentially confirm the factor structure detected in the EFA, as using the same sample of 120 individuals for both EFA and CFA is considered an undesirable practice (Lorenzo-Seva, 2022). Therefore, additional research is necessary to explore the factor structure of the revised *MISSCARE Survey* in Norway.

### Implications for Practice

This study has several clinical and organizational implications. The Norwegian version of the revised *MISSCARE Survey* can be used to identify areas where nursing care is missed in medical and surgical departments, thereby improving patient care and monitoring care quality. The survey may be used to guide hospitals in allocating resources more efficiently to areas that need them the most and identify the need for required support. The instrument can also be used to further investigate the quality of communication and collaboration, which may enhance teamwork and coordination among care personnel, ultimately leading to better patient outcomes. Finally, having an instrument that can identify critical work overload or care burden can be used to implement strategies to manage workload more effectively, ensuring that nurses are not overburdened and can provide high-quality care. Nonetheless, further studies should be conducted to confirm the factor structure in larger and more diverse populations.

## Conclusion

This study found that the Norwegian adaptation of the revised *MISSCARE Survey* is a promising instrument for assessing missed nursing care in medical and surgical departments within Norwegian hospitals. The EFA revealed that Part B of the Norwegian version of the revised *MISSCARE Survey* comprises a four-factor structure and that the instrument demonstrates good internal consistency. One factor represents the quality and level of external resources, another is about required support, the third focuses on the quality of communication and collaboration, and the fourth is about critical work overload or care burden.

## Supplemental Material

sj-docx-1-son-10.1177_23779608251332742 - Supplemental material for Translating, Adapting and Validating the Revised MISSCARE Survey for Use in Norwegian Hospitals—A Pilot Study Supplemental material, sj-docx-1-son-10.1177_23779608251332742 for Translating, Adapting and Validating the Revised MISSCARE Survey for Use in Norwegian Hospitals—A Pilot Study by Kjersti Grønning, Melliane Muteba Olsen and Beate André in SAGE Open Nursing

sj-docx-2-son-10.1177_23779608251332742 - Supplemental material for Translating, Adapting and Validating the Revised MISSCARE Survey for Use in Norwegian Hospitals—A Pilot Study Supplemental material, sj-docx-2-son-10.1177_23779608251332742 for Translating, Adapting and Validating the Revised MISSCARE Survey for Use in Norwegian Hospitals—A Pilot Study by Kjersti Grønning, Melliane Muteba Olsen and Beate André in SAGE Open Nursing
