# Supplementary material for: Translating, Adapting and Validating the Revised MISSCARE Survey for Use in Norwegian Hospitals—A Pilot Study
Source: SAGE Open Nurs. 2025 Apr 4;11:23779608251332742. doi: 10.1177/23779608251332742 (PMC11970074; doi:10.1177/23779608251332742)
Supplement: sj-docx-1-son-10.1177_23779608251332742 - Supplemental material for Translating, Adapting and Validating the Revised MISSCARE Survey for Use in Norwegian Hospitals—A Pilot Study [file sj-docx-1-son-10.1177_23779608251332742.docx]

**Modifications of the Norwegian version of the revised *MISSCARE Survey***

| Background items | | Part A items | | Part B items | |
| --- | --- | --- | --- | --- | --- |
| 3 | Adapted to Norwegian levels of education | 5 | Modified to Norwegian guidelines | 15 | Rephrased and added examples of different roles that work as nursing assistants in Norway |
| 7 | Adapted to Norwegian titles /job roles | 6 | Added examples of Vital signs | 16 | Gave examples of caregivers off the unit or unavailable |
| 9 | Adapted to Norwegian work hours | 12 | Added “twice a day” according to Norwegian guidelines |  |  |
| 12 | Adapted to Norwegian work shifts | 13 | Added “staff” |  |  |
|  |  | 14 | Rephrased /added “providing information to the patient” |  |  |
|  |  | 22 | Rephrased into attending interdisciplinary meetings |  |  |
|  |  | 23 | added “WC or bedpan” |  |  |
|  |  | 24 | Changed to wound care |  |  |
|  |  | Response alternative | Added “not relevant” |  |  |

The revised version of the *MISSCARE* *Survey* is described in the following paper: *Dabney, B. W., Kalisch, B. J., & Clark, M. (2019). A revised MISSCARE survey: Results from pilot testing. Appl Nurs Res, 50, 151202. DOI:*[*10.1016/j.apnr.2019.151202*](https://doi.org/10.1016/j.apnr.2019.151202)*.*
