# Supplementary material for: Translating, Adapting and Validating the Revised MISSCARE Survey for Use in Norwegian Hospitals—A Pilot Study
Source: SAGE Open Nurs. 2025 Apr 4;11:23779608251332742. doi: 10.1177/23779608251332742 (PMC11970074; doi:10.1177/23779608251332742)
Supplement: sj-docx-2-son-10.1177_23779608251332742 - Supplemental material for Translating, Adapting and Validating the Revised MISSCARE Survey for Use in Norwegian Hospitals—A Pilot Study [file sj-docx-2-son-10.1177_23779608251332742.docx]

**MISSED NURSING CARE (*The MISSCARE Survey*)**

Beatrice J. Kalisch


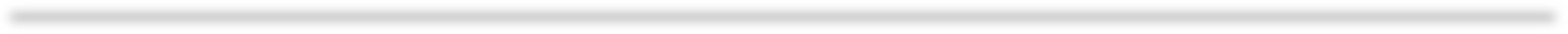


1. **Name of the unit** you work on:
2. I spend **the majority of my working time** on this unit**:** yes no

# Highest education level:

- 1. Grade school
  2. High School Graduate (or GED)
  3. Associate degree graduate
  4. Bachelor’s degree graduate
  5. Graduate degree

# If you are a nurse, what is the highest degree:

- 1. LPN Diploma
  2. RN Diploma
  3. Associate’s degree in nursing (ADN)
  4. Bachelor’s degree in nursing (BSN)
  5. Bachelor’s degree **outside** of nursing
  6. Master’s degree (MSN) or higher in nursing
  7. Master’s degree or higher **outside** of nursing

1. **Gender:** Female Male

# Age:

- 1. Under 25 years old (<25)

2) 25 to 34 years old (25-34)

3) 35 to 44 years old (35-44)

4) 45 to 54 years old (45-54)

5) 55 to 64 years old (55-64)

6) Over 65 years old (65+)

# Job Title/Role:

- 1. Staff Nurse (RN)
  2. Staff Nurse (LPN)
  3. Nursing Assistant (e.g., nurse aides/tech)
  4. Nurse manager, assistant manager (e.g. administrators on the unit)
  5. Other [Please specify: ]

***Please turn over to page 2*** 🡺

1. Number of **hours usually worked per week** (check only one)
   1. less than 30 hours per week
   2. 30 hours or more per week
2. **Work hours** (check the one that is most descriptive of the hours you work)
   1. Days (8 or 12 hour shift)
   2. Evenings (8 or12 hour shift)
   3. Nights (8 or 12 hour shift)
   4. Rotates between days, nights or evenings

# Experience in your role:

- 1. Up to 6 months
  2. Greater than 6 months to 2 years
  3. Greater than 2 years to 5 years
  4. Greater than 5 year to 10 years
  5. Greater than 10 years

# Experience on your current patient care unit:

- 1. Up to 6 months
  2. Greater than 6 months to 2 years
  3. Greater than 2 years to 5 years
  4. Greater than 5 year to 10 years
  5. Greater than 10 years

1. Which **shift** do you most often work?
   1. 8 hour shift
   2. 10 hour shift
   3. 12 hour shift
   4. 8 hour and 12 hour rotating shift
   5. Other [Please specify: ]
2. In the past 3 month, how many hours of **overtime** did you work?
   1. None
   2. 1-12 hours
   3. More than 12 hours
3. In the past 3 months, how many days or shifts did you **miss work** due to illness, injury, extra rest etc. (exclusive of approved days off)?
   1. None
   2. 1 day or shift
   3. 2-3 days or shifts

***Please turn over to page 3*** 🡺

- 1. 4-6 days or shifts
  2. over 6 days or shifts

1. Do you plan to **leave your current position?**
   1. in the next 6 months
   2. in the next year
   3. no plans to leave
2. How often do you feel **the unit staffing is adequate?**
   1. 100% of the time
   2. 75% of the time
   3. 50% of the time
   4. 25% of the time
   5. 0% of the time
3. **On the current or last shift** you worked, how many **patients** did you care for?

17-a. how many **patient-admissions** did you have (i.e. includes transfers into the unit)?

17-b. how many **patient-discharges** did you have (i.e. includes transfers out of the unit)?

**Please check** one **response for each question.**

|  | **Very satisfied** | **Satisfied** | **Neutral** | **Dissatisfied** | **Very dissatisfied** |
| --- | --- | --- | --- | --- | --- |
| 18. **How satisfied** are you in your **current position**? |  |  |  |  |  |
| 19. Independent of your current job, **how satisfied** are you with **being a nurse or a nurse assistant?** |  |  |  |  |  |
| 20. **How satisfied** are you with **the level of teamwork on this unit?** |  |  |  |  |  |

***Please turn over to page 4*** 🡺

| 4 of 6  **Section A — Missed Nursing Care**  Nurses frequently encounter multiple demands on their time, requiring them to reset priorities, and not accomplish all the care needed by their patients. To the best of your knowledge, **how frequently** are the following elements of **nursing care MISSED by the nursing staff (including you) on your unit? *Check only one box for each item.*** | | | | | | |
| --- | --- | --- | --- | --- | --- | --- |
|  |  | **Always**  missed | **Frequently**  missed | **Occasionally**  missed | **Rarely**  missed | **Never**  missed |
|  | **1) Ambulation/mobilization three times per day or as ordered** |  |  |  |  |  |
|  | **2) Turning patient every 2 hours** |  |  |  |  |  |
|  | **3) Feeding patient when the food is still warm** |  |  |  |  |  |
|  | **4) Setting up meals for patient who feeds themselves** |  |  |  |  |  |
|  | **5) Medications administered within 30 minutes before or after scheduled time** |  |  |  |  |  |
|  | **6) Vital signs assessed as ordered** |  |  |  |  |  |
|  | **7) Monitoring intake/output** |  |  |  |  |  |
|  | **8) Full documentation of all necessary data** |  |  |  |  |  |
|  | **9) Patient teaching about illness, tests, and diagnostic studies** |  |  |  |  |  |
|  | **10) Emotional support to patient and/or family** |  |  |  |  |  |
|  | **11) Patient bathing/skin care** |  |  |  |  |  |
|  | **12) Mouth care** |  |  |  |  |  |
|  | **13) Hand washing** |  |  |  |  |  |
|  | **14) Patient discharge planning and teaching** |  |  |  |  |  |
|  | **15) Bedside glucose monitoring as ordered** |  |  |  |  |  |
|  | **16) Patient assessments performed each shift** |  |  |  |  |  |
| ***Please turn over to page 5*** 🡺  @All rights protected Beatrice Kalisch  Please do not reproduce without permission of the author. Revised January 9, 2019 | | | | | | |

| 5 of 6 | | | | | | |
| --- | --- | --- | --- | --- | --- | --- |
|  |  | **Always**  missed | **Frequently**  missed | **Occasionally**  missed | **Rarely**  missed | **Never**  missed |
|  | **17) Focused reassessments according to patient condition** |  |  |  |  |  |
|  | **18) IV/central line site care and assessments according to hospital policy** |  |  |  |  |  |
|  | **19) Response to call light is initiated within 5 minutes** |  |  |  |  |  |
|  | **20) PRN medication requests acted on within 15 minutes** |  |  |  |  |  |
|  | **21) Assess effectiveness of medications** |  |  |  |  |  |
|  | **22) Attend interdisciplinary care conferences whenever held** |  |  |  |  |  |
|  | **23) Assist with toileting needs within 5 minutes of request** |  |  |  |  |  |
|  | **24) Skin/Wound care** |  |  |  |  |  |
|  | **25) Adequate surveillance of confused/impaired patients** |  |  |  |  |  |
| **Section B—Reasons for Missed Nursing Care**  Thinking about the missed nursing care on your unit by all of the staff (as you indicated on Part 1 of this survey), indicate the **REASONS nursing care is MISSED** on your unit. ***Check only one box for each item.***  @All rights protected Beatrice Kalisch  Please do not reproduce without permission of the author. Revised January 9, 2019 | | | | | | |

|  | **Significant**  reason | **Moderate**  reason | **Minor**  reason | NOT a  reason for missed care |
| --- | --- | --- | --- | --- |
| **1) Inadequate number of staff** |  |  |  |  |
| **2) Urgent patient situations (e.g. a patient’s condition worsening)** |  |  |  |  |
| **3) Unexpected rise in patient volume and/or acuity on the unit** |  |  |  |  |
| **4) Inadequate number of assistive and/or**  **clerical personnel (e.g. nursing assistants, techs, unit secretaries etc.)** |  |  |  |  |
| **5) Unbalanced patient assignments** |  |  |  |  |

***Please turn over to page 6*** 🡺

|  | **Significant**  reason | **Moderate**  reason | **Minor**  reason | **NOT** a  reason for missed care |
| --- | --- | --- | --- | --- |
| **6) Medications were not available when needed** |  |  |  |  |
| **7) Inadequate hand-off from previous shift or sending unit** |  |  |  |  |
| **8) Other departments did not provide the care needed (e.g. physical therapy did not ambulate)** |  |  |  |  |
| **9) Supplies/ equipment not available when needed** |  |  |  |  |
| **10) Supplies/ equipment not functioning properly when needed** |  |  |  |  |
| **11) Lack of back up support from team members** |  |  |  |  |
| **12) Tension or communication breakdowns with other ANCILLARY/SUPPORT DEPARTMENTS** |  |  |  |  |
| **13) Tension or communication breakdowns within the NURSING TEAM** |  |  |  |  |
| **14) Tension or communication breakdowns with the MEDICAL STAFF** |  |  |  |  |
| **15) Nursing assistant did not communicate that care was not provided** |  |  |  |  |
| **16) Caregiver off unit or unavailable** |  |  |  |  |
| **17) Heavy admission and discharge activity** |  |  |  |  |
| **18) Emotional or physical exhaustion** |  |  |  |  |
| **19) Inadequate supervision of nursing assistants** |  |  |  |  |
| **20) Interruptions/Multitasking** |  |  |  |  |
| **21) Lack of cues/reminders** |  |  |  |  |
| **22) Inadequate support from leadership** |  |  |  |  |

**THANK YOU FOR YOUR PARTICIPATION!**
